# Correction: Core/shell upconversion nanoparticles with intense fluorescence for detecting doxorubicin *in vivo*

**DOI:** 10.1039/c8ra90055h

**Published:** 2018-07-02

**Authors:** Junshan Hu, Shiping Zhan, Xiaofeng Wu, Shigang Hu, Shaobing Wu, Yunxin Liu

**Affiliations:** Department of Physics and Electronic Science, Hunan University of Science and Technology China lyunxin@163.com; Department of Information Science, Hunan University of Science and Technology Xiangtan 411201 China xfwuvip@126.com

## Abstract

Correction for ‘Core/shell upconversion nanoparticles with intense fluorescence for detecting doxorubicin *in vivo*’ by Junshan Hu *et al.*, *RSC Adv.*, 2018, **8**, 21505–21512.

The authors regret that some of the affiliation and contact details were incorrect in the original manuscript. The correct affiliation and contact details are shown above.

The Royal Society of Chemistry apologises for these errors and any consequent inconvenience to authors and readers.

## Supplementary Material

